# The effects of locking inserts and overtorque on the mechanical properties of a large fragment locking compression plate

**DOI:** 10.1186/s40634-021-00424-0

**Published:** 2021-11-24

**Authors:** Kathleen N. Meyers, Timothy S. Achor, Mark L. Prasarn, Jaimo Ahn, Kevin Khalsa, David S. Wellman, Dean G. Lorich, David L. Helfet

**Affiliations:** 1 Department of Biomechanics, Hospital for Special Surgery, New York, NY USA; 2 Orthopaedic Trauma Service, Hospital for Special Surgery, New York Presbyterian Hospital, Weill Cornell Medicine, 535 East 70th Street, New York, NY 10021 USA; 3 Orethopaedic Trauma Service, Westchester Medical Center, New York Medical College, Valhalla, NY USA

**Keywords:** Locking inserts, Overtorque, Mechanical testing, Mechanical properties, Locking plate, Locking compression plate

## Abstract

**Purpose:**

The study was to determine the effect of locking hole inserts and their insertion torque on the fatigue life of a large fragment Locking Compression Plate (LCP) under bending forces.

**Methods:**

Fatigue strength of the LCP was examined using cyclic three-point bend testing at 80% yield strength of the construct. Locking hole inserts were used in 2, 4, and 6-hole of a 12-hole plate to simulate three different working lengths. Within each working length, plates were tested without locking inserts serving as the control group. In the experimental groups, inserts were tightened to manufacturer recommendations (4 Nm) and using overtorque (8 Nm).

**Results:**

Significantly fewer cycles to failure were observed in control groups versus the locking hole insert groups for all working lengths (2-hole: 4 Nm *p* = 0.003, 8 Nm *p* = 0.003; 4-hole: 4 Nm *p* = 0.02, 8 Nm *p* < 0.001; 6-hole: 4 Nm *p* = 0.004, 8 Nm *p* < 0.001). There was a statistically significant increase in fatigue strength when using overtorque in the 4-hole (*p* = 0.04) and 6-hole (*p* = 0.01) defect groups. This was not shown in the 2-hole defect group (*p* = 0.99).

**Conclusions:**

By placing locking inserts in the empty locking regions of Combi holes along the working length, the number of cycles to failure was increased. Tightening inserts to twice the recommended insertion torque further increased cycles to failure in longer working length models. A longer fatigue life has the potential to decease the incidence of plate failure especially in the setting of delayed union due to poor intrinsic healing capacity, fractures in the geriatric population, osteoporosis and periprosthetic fractures.

## Background

The locking compression plate (LCP; Synthes, Paoli, PA) is an implant which allows use of both conventional screw fixation and locking screw fixation via Combi holes in the plate that have regions for both screw types within the same hole. Locking plate technology has been advocated for use in osteoporotic bone, minimally invasive osteosynthesis, and complex periarticular fractures [1]. As a result, locked plating has gained popularity amongst orthopaedic surgeons for use in fracture care [5, 8, 10, 14, 15]. The LCP offers various anchoring options, allowing the surgeon to use conventional, locking, or hybrid screw insertion techniques. This property allows traditional dynamic compression plating and the ability to create a fixed-angle construct within the same device.

The stability and biomechanical properties of large fragment locking plate constructs have been studied and well described. The working length (the distance of the first screw to the fracture site), the number of screws, and the distance of the plate to the bone are all known to effect the axial stiffness and torsional rigidity of the construct [16]. Locking hole inserts, also known as threaded screw head inserts, have been evaluated to determine whether or not filling the empty locking holes changes the properties of the plate once all screws have been placed. Cartner et al. determined the fatigue properties of a locking plate with round locking holes could be improved by using locking inserts in the empty holes [4]. Bellapianta et al. studied the effects of locking screw heads on the biomechanical properties of locking plates. Using one third tubular plates from two different manufacturers, they found the fatigue life as well as stiffness could be increased by including locking inserts in the constructs tested [3]. A prior study by Zhang et al. in a mechanical study found that locking hole inserts, applied to 3.5 mm locking plates with round locking holes (without Combi holes) extended the cycles to failure by 52% compared to those without locking hole inserts, and a 48% increase in plate cycles to failure when locking hole inserts were applied to 4.5 mm locking plates with round locking holes (without Combi holes) compared to those without locking hole inserts [19]. This study also found that increasing insertion torque from manufacturer’s recommended 1.70 Nm to 3.96 Nm led to a further 36% increase in cycles to failure. As the locking insert in the Synthes LCP only fills only part of the Combi hole, the effects are potentially different from previously tested constructs [7].

Further, it has been shown in the engineering literature that over-torque applied to conical threaded connections results in improved performance in terms of static and fatigue resistance of the connection [2]. The authors of this study specifically looked at the axial dynamic fatigue load effects on friction behavior during makeup of threaded connections. Using experimental tests and numerical simulations they were able to show that there was a favorable thread load distribution produced by overloading with increased torque [2]. It is unclear what effect over-torque may have on the biomechanical properties of locking plates used for osteosynthesis. Furthermore, it is unknown how much additional torque can be imparted by the surgeon who is manually tightening the screws.

There is potential clinical applicability of the locking inserts if they are found to offer beneficial mechanical properties. In the clinical setting, this could be useful in patients where plate breakage is a significant concern. Screw and plate breakage have been reported with use of the LCP, in particular with bridge plating of comminuted fractures or when the patient poses a poor biological environment for fracture healing. Potential methods of improving the mechanical properties of the plate should therefore be explored as a possible way to decrease fixation failures. Considering the increased prevalence and use of plates with Combi holes, the purpose of this study is to determine: (i) if the fatigue resistance of an LCP with Combi holes can be improved by filling the unused locking portion of the holes with locking inserts for different working length plate constructs and (ii) the effect of insertion torque for the locking inserts on the fatigue life of these plate constructs. We hypothesize that adding locking inserts will increase the fatigue life of the LCP and that torqueing the insert to higher level will increase fatigue life even further.

## Methods

**Fig. 1:**
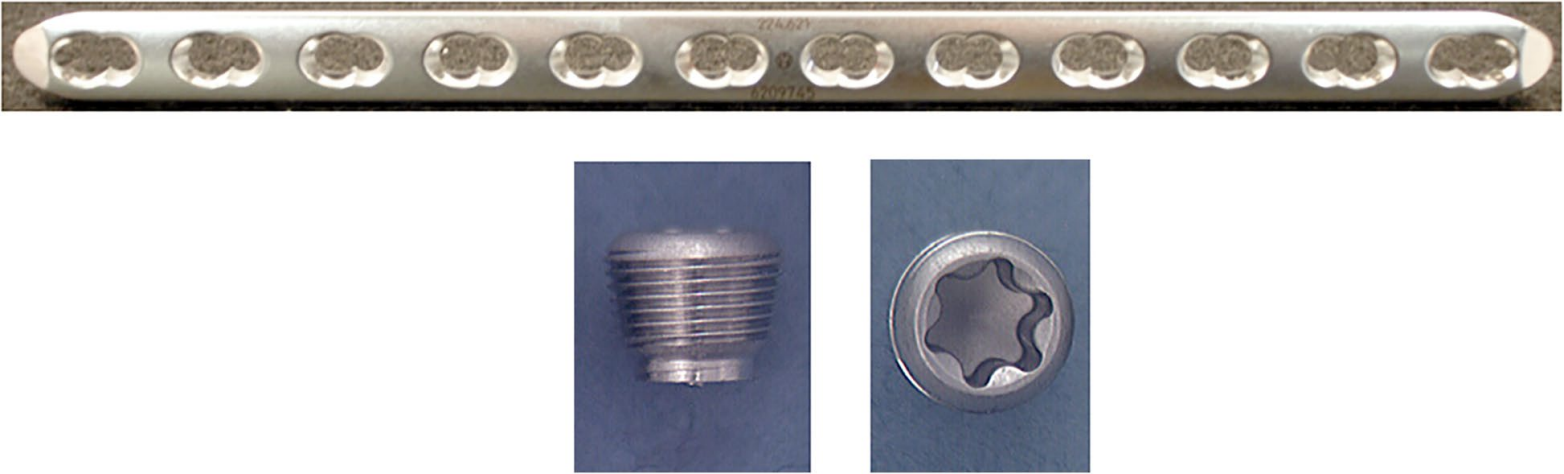
Standard narrow 4.5 mm large fragment Locking Compression Plates (LCP) with Combi holes were used for all tests (top image). Locking inserts (bottom images) are similar in design to locking screws comprise only the upper aspect of the locking screw for threaded engagement with the locking plate

**Fig. 2:**
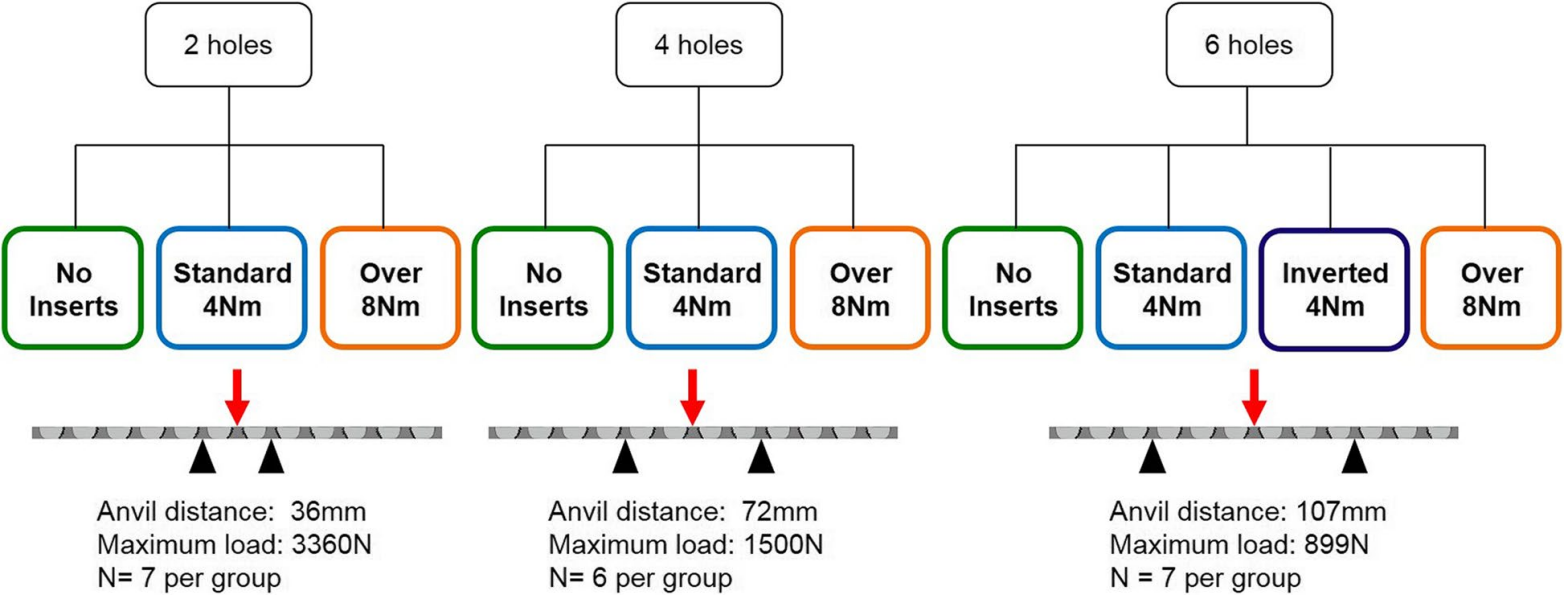
Flowchart detailing the experimental protocol and study groups

Standard stainless steel 12-hole narrow 4.5 mm large fragment Locking Compression Plates with Combi holes (DePuy Synthes, Paoli, PA) were used for all tests along with locking inserts (Depuy Synthes, Paoli, PA) in varying testing configurations, as described below (Fig. 1). We tested 2, 4, and 6-hole defects to simulate different working lengths. Within these three working lengths, plates were tested without locking inserts (Controls), with locking inserts tightened to 4 Nm (Normal torque), and with locking inserts tightened to 8 Nm (0vertorque). 2 and 6-hole constructs were tested first to represent the extremes of gap length. Seven of each plate configuration was tested to determine if there was an effect of working length. A post hoc sample size analysis of the 2- and 6-holes tests was conducted to determine if more samples were needed and to set the number of plates to use in subsequent testing to achieve at least 80% with significance set at *p* < 0.05(*n* = 6 each). In addition, the 6-hole defect with inserts at normal torque was tested with the plate inverted to ensure that the direction of plate bending did not affect construct strength; (Fig. 2). A total of 66 plates were cyclically tested.

The manufacturer recommends a 4 Nm screw insertion torque when using locking screws in large fragment plates. This was defined as the normal insertion torque for this study. Pilot testing was completed to determine what insertion torque would be used for the overtorque configurations. Maximal torque manually produced by 7 orthopaedic surgeons was measured using an AWS-QC torque tester (Advanced Witness Series, INC, San Jose, CA, US). The dominant extremity was used while performing all tests. The average recorded torque was 6.9 Nm (range 5.9-8.3 Nm). The maximum of these readings, 8 Nm (twice the torque level as recommended by the manufacturer), was then defined as overtorque and used in all test samples. A torque limiting driver set to either 4 Nm or 8 Nm was used for all screw head insertions.

Pilot testing was also performed to determine load levels for cyclic testing. The yield strength of 9 plates of 2-hole working length without inserts (*n* = 3), with inserts at normal torque (*n* = 3), and with inserts at overtorque (*n* = 3) was determined by loading each construct to failure in three-point bend (50 N/s). Since the yield strength of the overtorque group was statistically higher than both the normal (*p* = 0.01) and no insert (*p* = 0.03) groups and there was no statistical difference between the no insert and normal torque group (*p* = 0.86), plates with 4-hole and 6-hole working lengths were loaded to failure without inserts only (*n* = 3 each). Maximum cyclic load levels were set at 80% of the yield strength.

Once insert torque levels and yield strength were determined, cyclic three-point bend testing was performed for each working length and insert combination. Plates were positioned such that support anvils spanned either 2, 4, or 6 holes to reflect the working length being tested and were cycled to failure at 80% yield strength (5 Hz) based on that specific working length (2-hole: 10-3360 N, 4-hole: 10-1500 N, 6-hole: 10-899 N, Fig. 2). Load and piston displacement data were collected throughout cycling. The number of cycles to construct failure was determined and the site of failure in the plate was noted for all constructs.

Statistical analysis comparing cycles to failure between constructs at 2-, 4-, and 6-hole working lengths was performed using one-way ANOVA with Holm-Sidak post hoc analysis with significance set as *p* < 0.05. Synthes (West Chester, PA, USA) provided all Locking Compression Plates and locking hole inserts for research use in this mechanical investigation. No external funding was received for this research investigation.

## Results

### Mode of failure

**Fig. 3:**
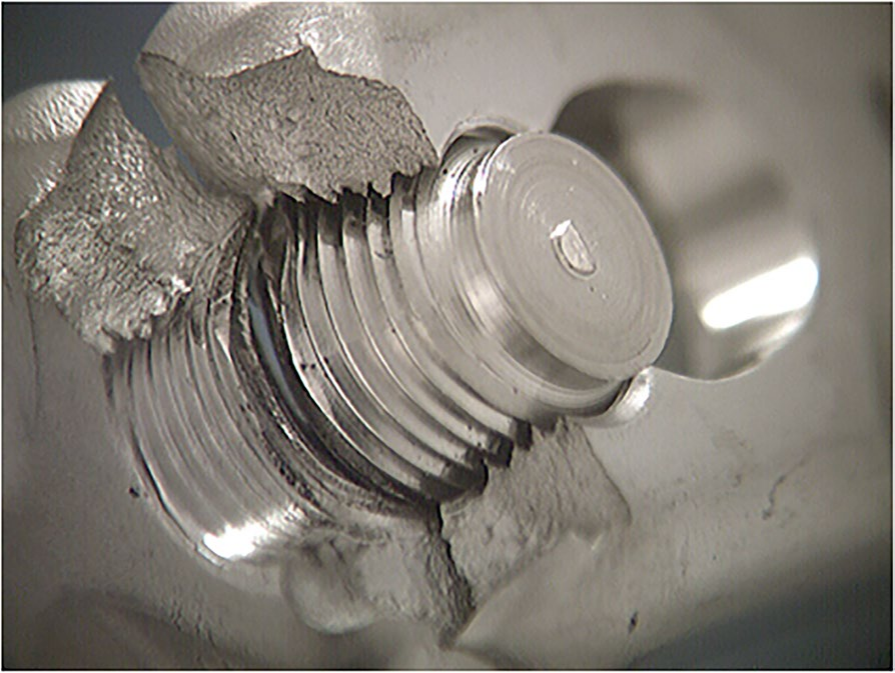
All plates failed through the threaded portion of a hole adjacent to the loading anvil

During the cyclic mechanical testing phase, all plates failed through the threaded portion of a hole adjacent to the loading anvil (Fig. 3).

### 2-hole constructs

**Fig. 4:**
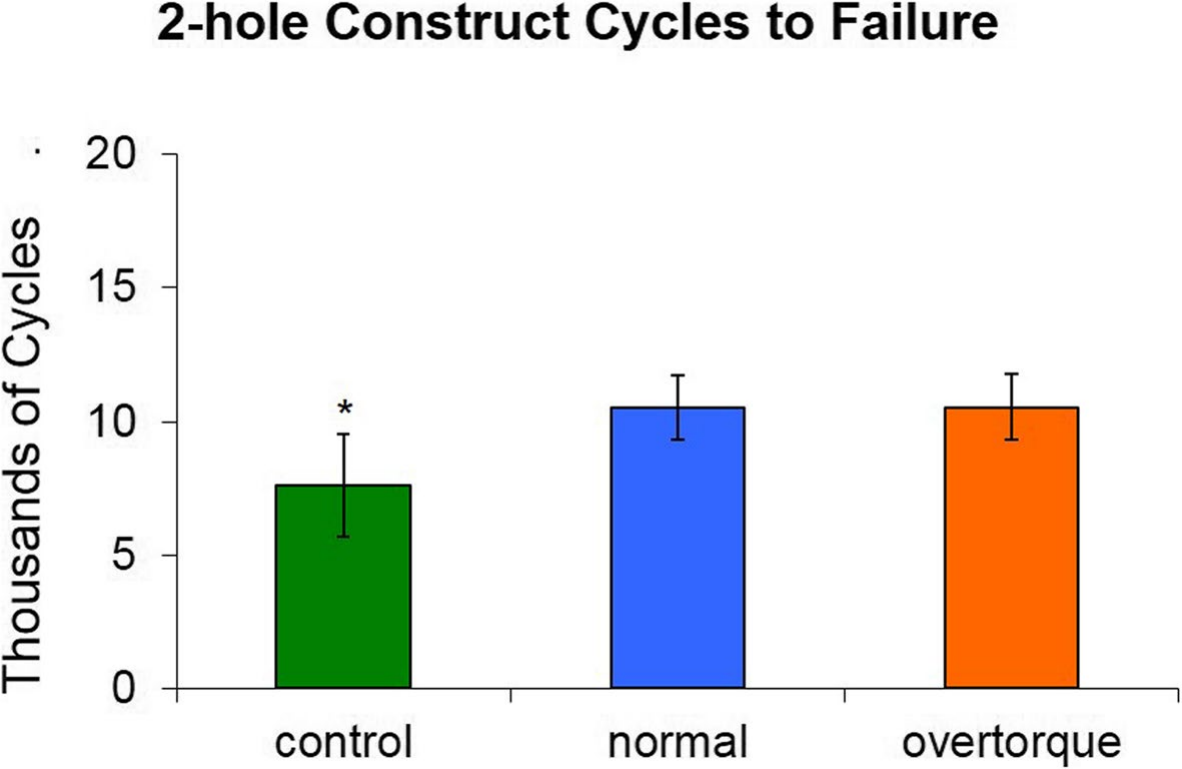
Average cycles to failure for two-hole constructs (mean ± standard deviation). Significantly fewer cycles were required to failure in the control group versus the normal and overtorque group. No difference was found between the normal and overtorque groups

Adding locking inserts to the 2-holes defect working length model increased the number of cycles to failure. This was demonstrated for both the 4 Nm (*p* = 0.003) and 8 Nm (*p* = 0.003) constructs as compared to controls. There was no observed difference in number of cycles to failure between the 4 Nm and 8 Nm groups (Fig. 4).

### 4-hole constructs

**Fig. 5:**
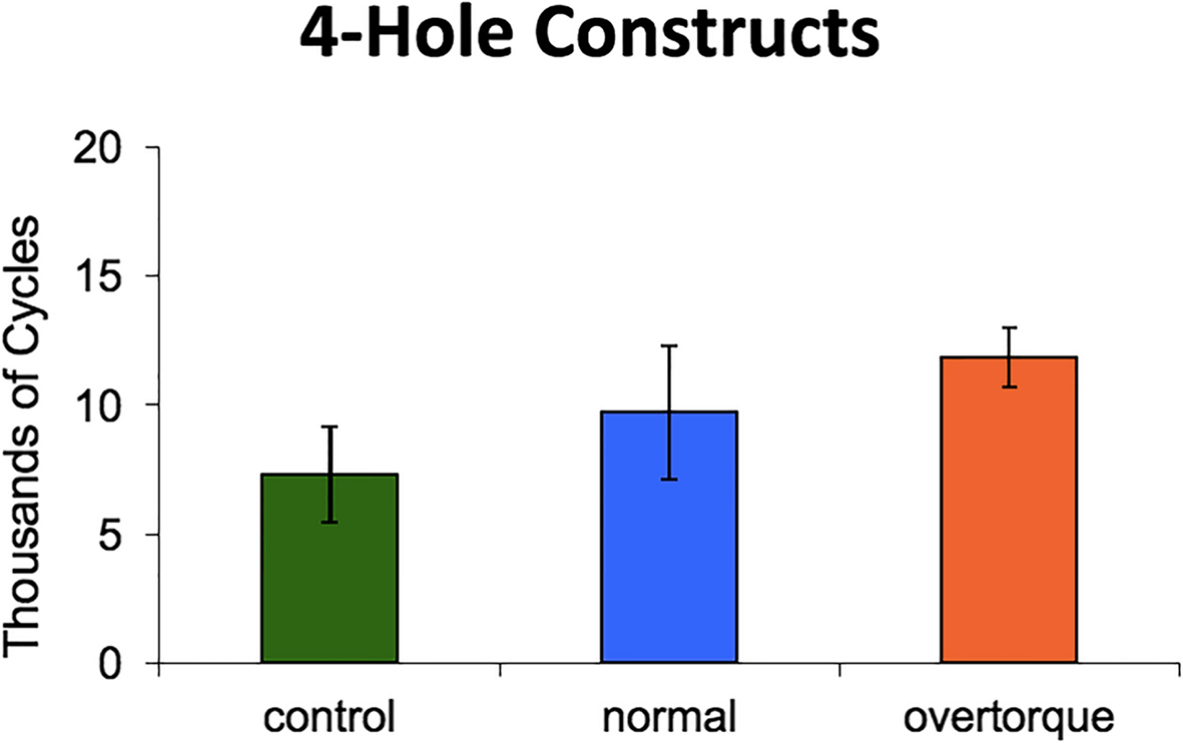
Average cycles to failure for four-hole constructs (mean ± standard deviation). Significantly fewer cycles were required to failure in the control group versus the normal and overtorque group. A significant difference was also found between the normal and overtorque groups

Within the 4-hole defect group, plate failure occurred at significantly fewer cycles in controls as compared to both the 4 Nm (*p* = 0.02) and 8 Nm (*p* < 0.001) locking insert groups. Overtorque did improve the strength of this construct. The 4 Nm group failed at significantly fewer cycles as compared to the 8 Nm group (*p* = 0.04); (Fig. 5).

### 6-hole constructs

The 6-hole defect group also showed that plate failure occurred at significantly fewer cycles in controls as compared to both the 4 Nm (*p* = 0.004) and 8 Nm (*p* < 0.001) locking insert groups. The 4 Nm group failed at significantly fewer cycles as compared to the 8 Nm group (*p* = 0.01).

### Effect of plate orientation during testing

**Fig. 6:**
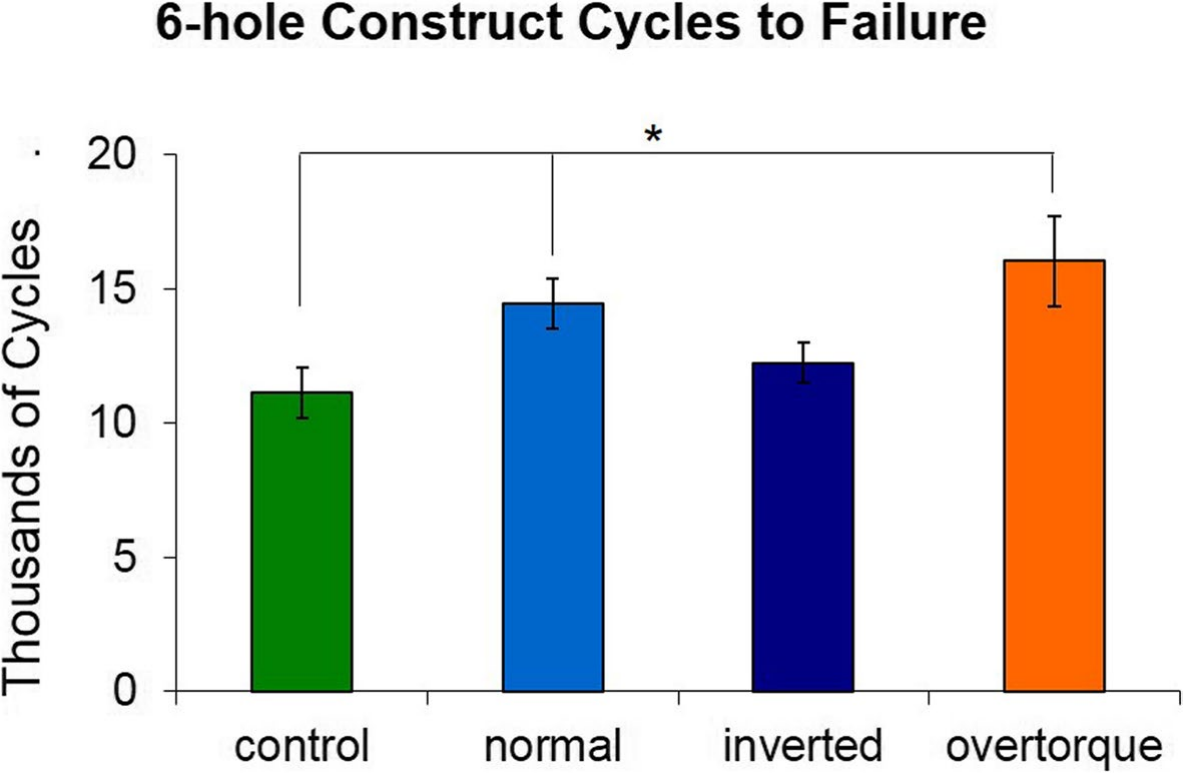
Average cycles to failure for six-hole constructs (mean ± standard deviation). The overtorque group had significantly more cycles to failure than control, normal, and inverted group. The control group also had significantly fewer cycles to failure that the normal torque group. The inverted group did not differ from the normal group

There was no statistical difference in number of cycles to failure when the 6-hole normal insert constructs were tested with the LCP inverted (Fig. 6). This construct had the longest working length and was therefore most likely to experience an effect when inverted. Since there was no difference with a 6-hole working length, the test was not repeated for 2- and 4-hole constructs.

## Discussion

The results of our study support our hypotheses by demonstrating that using locking inserts in what would otherwise be empty holes increases the fatigue strength of a stainless steel large fragment LCPs in different working length constructs in a mechanical testing environment. Furthermore, by tightening the inserts to twice the recommended insertion torque, the fatigue strength can be further increased in longer working length models. It was also shown that this amount of torque is potentially reproducible in the clinical setting by the average orthopaedic surgeon. A specialized driver could be developed to ensure an 8 Nm torque is applied to the inserts. This may have clinical applications where bridge plating has left several threaded holes open and the potential for fixation failure (plate breakage) exists.

A known mechanism of treatment failure with the large fragment LCP is plate breakage, typically the result of repeated bending loads. Clinically this failure typically occurs through empty holes in the plate. It occurs most commonly where longer segments of comminution are spanned and in the epimetaphyseal region. Sommer et al. reported on an initial prospective series of 169 fractures in a heterogenous group of 144 patients treated with the LCP. Fracture healing was observed in 86%, and there were 9 fixation failures [15]. The same authors later reported on four cases of LCP loosening and breakage. They state that a stronger plate may have withstood the load sharing for a longer period, potentially preventing breakage and treatment failure [14].

Our findings are consistent with prior reports in the literature and provide further data on the mechanical properties of locking plates with inclusion of locking inserts in both standard configuration and over-torque configuration in a Combi hole. In a study by Cartner et al., 3.5 mm and 4.5 mm locking plates were tested with locking hole inserts for both stiffness and fatigue mechanical properties [4]. The authors found increased cycles to failure for both plate lengths with the presence of locking hole inserts. Bellapianta et al. also assessed the stiffness and fatigue characteristics of presence of locking screw heads in empty screw holes of locking plates using finite element analysis followed by an experimental synthetic bone model using four-point bending [3]. Finite element analysis results showed filling of empty plate holes resulted in decreased maximum stress at the periphery of the holes. Mechanical testing results revealed significant improvement in fatigue life in all plates tested.

Tompkins et al. performed a study with synthetic femurs and locking plate construct groups (8-hole plates) in a segmental bone defect. The four experimental groups consisted of (1) all locking screws (*n* = 5), (2) all compression screws (*n* = 5), (3) six compression screws with two locking buttons in the central holes (*n* = 6), and (4) six compression screws with two open central holes (*n* = 6) [18]. The authors found that the button group had the longest fatigue life with no differences found between the locked and unlocked groups. This compares positively with our finding that fewer cycles to failure were observed in control groups versus the locking hole insert groups for all working lengths.

Not all studies have found significant differences for locking inserts. Firoozabadi et al. performed a study on Combi hole plates using a segmental bone defect model (6 cm metaphyseal defect) using multiple constructs including (1) unplugged, (2) plugged with locking screw only, and (3) fully plugged (using a custom form-fitting press-fit plug) [7]. They did not find a difference a between the constructs in fatigue life, axial or torsional stiffness. These findings are in contrast to our results which found fewer cycles to failure in control groups versus the locking insert groups at all working lengths. Eichinger et al. also performed a study using a 1 cm simulated segmental bone defect model with ^**1**^/_**3**_ tubular locking plates. In one group ten plates had a screw hole insert in the center hole (next to simulated fracture) and in the second group the center hole remained empty (next to the simulated fracture) [6]. No differences were found in torsional and axial stiffness or compressive strength to load to failure. Our study, which performed cyclic loading to failure at 80% yield strength based on specific working lengths, allowed for more discrete differences to be observed in fatigue; but clinical significance of these possible differences needs further study.

This study supports the clinical applicability of the use of locking inserts. We found that with placement of locking inserts into empty holes along the working length of an LCP, the number of cycles to failure was increased. A prolonged time to osseus union following fracture has been reported in multiple clinical scenarios, including in the elderly population [11], periprosthetic fractures and nonunions [13], and fractures associated with long-term alendronate therapy [12]. An increase in fatigue strength with the use of the LCP may also be of benefit in osteoporotic patients [17] or in fractures where there may be difficulty in obtaining adequate bony apposition (including those associated with significant bone loss or comminution) [9].

We do not know clinical implications of overtightening the locking inserts or screws. Attempting to remove locking screws or inserts that have been over tightened or “cold-welded” can prove to be a technical challenge for the orthopaedic surgeon while in the operating room. We did not attempt to remove any of the locking inserts. However, it should also be noted that removing them for fixation construct removal purposes would be unnecessary in clinical practice.

It is also important to note that a stiffer construct may not be beneficial in all cases. Fracture healing requires an optimal strain environment for the bone to heal appropriately. Overly stiff fixation constructs can lead to atrophic nonunions in some cases, and therefore, surgeons should be cognizant of the biomechanical effects that increasing the stiffness of their chosen plate may have upon the local strain environment at the fracture site.

The use of in vitro testing conditions is a limitation of this study. Clinically, plates are subject to a complex combination of axial loading, bending, and torsion. We tested in bending since this was seen as the dominant loading mode. An additional limitation to the present study is the lack of investigation into the effect of locking inserts on titanium plates or other plate designs. This may lead to different results than the current study demonstrates.

This study provides more evidence that locking inserts alter the properties of plates used in osteosynthesis, in particular the stainless steel large fragment LCP Combi hole. In addition, we have examined the effect of increased torque on locking inserts. The increase in fatigue stength by both the addition of inserts and over-torquing of locking inserts have the potential to decrease plate fracture prior to bone healing. The clinical implications of placing locking inserts in empty holes and using overtorque needs further investigated.

## Data Availability

The datasets used and/or analyzed during the current study are available from the corresponding author on reasonable request.
